# Longitudinal mortality of preserved ratio impaired spirometry in a middle-aged Asian cohort

**DOI:** 10.1186/s12890-023-02451-2

**Published:** 2023-05-03

**Authors:** Sooim Sin, Eun Ju Lee, Sungho Won, Woo Jin Kim

**Affiliations:** 1 Division of Pulmonary and Critical Care Medicine, Department of Internal Medicine, National Medical Center, Seoul, Republic of Korea; 2 Department of Internal Medicine and Environmental Health Center, Kangwon National University Hospital, Chuncheon, Republic of Korea; 3 Interdisciplinary Program in Bioinformatics, Seoul National University, Seoul, Republic of Korea; 4 Department of Public Health Science, Graduate School of Public Health, Seoul National University, Seoul, Republic of Korea; 5 Department of Internal Medicine, School of Medicine, Kangwon National University, Chuncheon, Republic of Korea; 6 Department of Internal Medicine, School of Medicine, Kangwon National University, Kangwon National University Hospital, Chuncheon, 24341 Republic of Korea

**Keywords:** Preserved ratio impaired spirometry, Mortality, Cardiovascular, Asian, Middle-aged

## Abstract

**Background:**

Although preserved ratio impaired spirometry (PRISm) has been determined to have poor prognosis, it is a heterogeneous state, and studies regarding its prognosis in Asians are limited. This study investigated the long-term all-cause and cardiovascular mortality of patients with PRISm compared with those of patients with chronic obstructive pulmonary disease (COPD) and normal individuals in the Korean middle-aged general population.

**Methods:**

Participants were recruited between 2001 and 2002 from a community-based prospective cohort in South Korea. Mortality data were collected over a 16.5-year mean follow-up period. The all-cause and cardiovascular mortality risks of PRISm were compared between patients with COPD and healthy controls.

**Results:**

The PRISm group had a mean age of 53.4 years and mean body mass index of 24.9 kg/m^2^; furthermore, 55.2% of the PRISm patients had never smoked, and the prevalence of comorbidities was not higher than that in the other groups. Compared with normal individuals, PRISm patients did not show increased all-cause mortality, whereas COPD patients showed increased all-cause mortality (PRISm: adjusted hazard ratio [aHR], 1.19; 95% confidence interval [CI], 0.85–1.65; COPD: aHR, 1.34, 95% CI, 1.07–1.69). Furthermore, the PRISm patients did not show increased cardiovascular mortality compared with normal individuals (PRISm: aHR, 1.65; 95% CI, 0.92–2.95; COPD: aHR, 1.83; 95% CI, 1.09–3.07).

**Conclusion:**

In our population-based cohort, all-cause and cardiovascular mortality risk did not increase in individuals with PRISm compared with normal individuals. Further studies are needed to distinguish a lower-risk subgroup of PRISm with certain characteristics, such as middle-aged, light-smoking Asians without additional cardiovascular risk.

**Supplementary Information:**

The online version contains supplementary material available at 10.1186/s12890-023-02451-2.

## Background

Preserved ratio impaired spirometry (PRISm), also known as restrictive spirometry, is defined as a reduced forced expiratory volume in 1 s (FEV_1_) with a normal FEV_1_/forced vital capacity (FVC) ratio that does not meet the criteria for chronic obstructive pulmonary disease (COPD) but cannot be considered normal [1]. Several studies were conducted on the disease course and prognosis of PRISm 15 years ago [2–5]. Most studies have confirmed that PRISm is associated with increased all-cause and cardiovascular mortality compared with individuals with normal spirometry [2, 6–11]. Accordingly, the importance of PRISm and its recognition in clinical practice are being increasingly emphasized. However, PRISm is considered a heterogeneous state, rather than a single mechanism, and thus has various risk factors [3, 5, 12–15]. Furthermore, the prevalence of PRISm varies greatly among different populations with widely varying ethnicities, ages, and clinical characteristics [5, 16–19]. The Rotterdam [10] and COPDGene studies [11] suggested that subgroups with higher or lower risk exist within PRISm. However, the prognosis of PRISm in various populations with different characteristics remains to be elucidated.

This study aimed to investigate the prevalence and prognosis of PRISm in COPD patients and individuals with normal lung function in a middle-aged Asian population.

## Methods

### Study design and population

In the present study, data were collected from the Korean Genome and Epidemiology Study (KoGES) Ansan and Ansung study. The KoGES Ansan and Ansung study is a prospective population-based cohort study supported by the Korean government. The cohort consisted of the general population aged ≥ 40 to ≤ 69 years at baseline. This study enrolled 10,030 participants recruited between 2001 and 2002. Details of the cohort have been published previously [20].

### Data collection and definitions

**Fig. 1:**
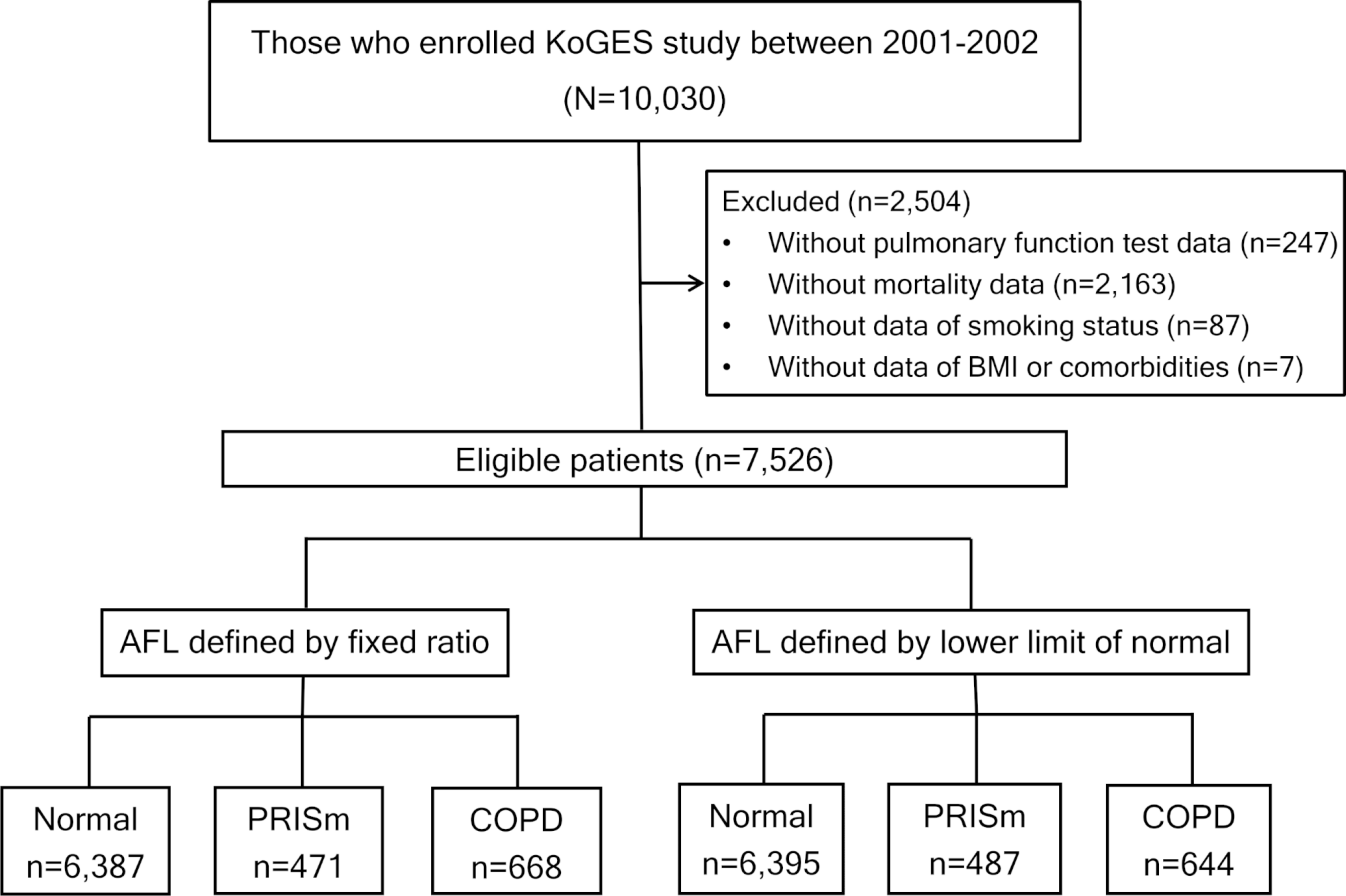
Flow diagram of the study population. AFL, airflow limitation; BMI, body mass index; COPD, chronic obstructive pulmonary disease; PRISm, preserved ratio of impaired spirometry

Among the baseline data of the cohort participants, smoking status (never, former, or current) was assessed using self-reported questionnaires, body mass index (BMI) was assessed through physical examination, and lung function was assessed by prebronchodilator spirometry. Among the enrolled participants, those without spirometry or smoking history data were excluded. Mortality analysis was performed using participants’ matched mortality data up to December 2018 from the Korean National Statistical Office. Participants without matched mortality data were excluded (Fig. 1).

Participants were classified as having COPD when they had airflow limitation (AFL), having PRISm when their percentage of predicted FEV_1_ was less than 80% without AFL, or normal [21]. AFL was defined as both the fixed ratio (FR) and lower limit of normal (LLN). The prediction equation for FEV1/FVC to estimate LLN was 125.77628−0.36304×age(11)−0.17146×height (cm) for male participants and 97.36197–0.26015×age(11)−0.01861×height (cm) for female participants [22–24].

### Statistical analysis

All analyses were performed twice by applying the AFL according to both the FR and LLN criteria. The one-way ANOVA was used for between-group comparisons of demographics involving continuous variables, and the chi-square test was used for those involving categorical variables. The Kaplan-Meier method was used for survival analysis to compare the all-cause mortality of the COPD, PRISm, and normal groups divided by each criterion. Cox proportional hazards regression analysis was used to present adjusted hazard ratios (aHRs) and their 95% confidence intervals (CIs). Statistical significance was set at two-sided P-values < 0.05. Statistical analyses were performed using SAS version 9.4 (SAS Institute, Cary, NC, USA).

## Results

### Prevalence of PRISm and baseline demographics of the study participants

A total of 7,526 participants were included in the analysis. The baseline demographic characteristics of the participants are presented in Table 1. Of the total participants, 8.8% had COPD and 6.2% had PRISm according to the FR criteria. According to the LLN criterion classification, 8.5% and 6.4% of patients had COPD and PRISm, respectively. Of the COPD patients, according to the FR criterion classification, 49.3% were in their 60s and 20.8% were in their 40s. In contrast, according to the LLN criterion classification, 32.3% were in their 60s and 39% were in their 40s. In the PRISm group, 42.5% were in their 40s and 27.8% were in their 60s according to the FR criteria. In contrast, 36.3% were in their 40s and 34.1% were in their 60s according to the LLN criteria. The COPD group had a higher proportion of male participants than the normal and PRISm groups, which was more prominent according to FR criteria. The PRISm group showed a higher proportion of participants with a high BMI than the other groups for both criteria.

**Table 1:** Baseline demographics of participants with normal spirometry, PRISm, and COPD

	Total (N = 7,526)	Fixed ratio				Lower limit of normal				
		Normal (n = 6,387)	PRISm (n = 471)	COPD (n = 668)	P-value		Normal (n = 6,395)	PRISm (n = 487)	COPD (n = 644)	P-value
Age (years)	51.9 ± 8.6	51.2 ± 8.5	53.4 ± 8.4	57.7 ± 8.1	< 0.001		51.5 ± 8.6	54.7 ± 8.6	53.8 ± 8.7	< 0.001
Age group					< 0.001					
40–49 years	3,659 (48.6)	3,320 (52.0)	200 (42.5)	139 (20.8)			3,231 (50.5)	177 (36.3)	251 (39.0)	
50–59 years	2,037 (27.1)	1,697 (26.6)	140 (29.7)	200 (29.9)			1,708 (26.7)	144 (29.6)	185 (28.7)	
60–69 years	1,830 (24.3)	1,370 (21.5)	131 (27.8)	329 (49.3)			1,456 (22.8)	166 (34.1)	208 (32.3)	
Male	3,597 (47.8)	2,858 (44.8)	248 (52.7)	491 (73.5)	< 0.001		2,913 (45.6)	271 (55.7)	413 (64.1)	< 0.001
BMI (kg/m^2^)	24.7 ± 3.1	24.7 ± 3.0	24.9 ± 3.5	23.7 ± 2.9	< 0.001		24.7 (3.1)	24.8 (3.5)	23.9 (3.0)	< 0.001
BMI group					< 0.001					< 0.001
< 18.5 kg/m^2^	115 (1.5)	80 (1.3)	17 (3.6)	18 (2.7)			79 (1.2)	18 (3.7)	18 (2.8)	
18.5–22.9 kg/m^2^	2,156 (28.7)	1,759 (27.5)	121 (25.7)	276 (41.3)			1,783 (27.9)	133 (27.3)	240 (37.3)	
23.0–24.9 kg/m^2^	1,979 (26.3)	1,714 (26.8)	92 (19.5)	173 (25.9)			1,717 (26.9)	92 (18.9)	170 (26.4)	
≥ 25.0 kg/m^2^	3,276 (43.5)	2,834 (44.4)	241 (51.2)	201 (30.1)			2,816 (44.0)	244 (50.1)	216 (35.5)	
Smoking status					< 0.001					< 0.001
Never	4,480 (59.5)	4,009 (62.8)	260 (55.2)	211 (31.6)			3,981 (62.3)	250 (51.3)	249 (38.7)	
Former	1,209 (16.1)	983 (15.4)	82 (17.4)	144 (21.6)			1,006 (15.7)	85 (17.5)	118 (18.3)	
Current	1,837 (24.4)	1,395 (21.8)	129 (27.4)	313 (46.9)			1,408 (22.0)	152 (31.2)	277 (43.0)	
Lung function										
FEV_1_% predicted	96.9 ± 13.9	100.4 ± 11.1	73.3 ± 6.1	80.2 ± 14.1	< 0.001		100.4 ± 11.1	73.4 ± 5.9	80.1 ± 14.3	< 0.001
FVC % predicted	96.8 ± 13.1	98.5 ± 11.4	73.6 ± 8.7	97.5 ± 14.9	< 0.001		98.4 ± 11.3	73.7 ± 8.5	98.7 ± 15.2	< 0.001
FEV_1_/FVC (%)	0.8 ± 0.1	0.8 ± 0.1	0.8 ± 0.1	0.6 ± 0.1	< 0.001		0.8 ± 0.1	0.8 ± 0.1	0.6 ± 0.1	< 0.001
Comorbidities										
HTN	1,112 (14.8)	917 (14.4)	95 (20.2)	100 (15.0)	0.002		927 (14.5)	105 (21.6)	80 (12.4)	< 0.001
DM	469 (6.2)	376 (5.9)	44 (9.3)	49 (7.3)	0.005		382 (6.0)	42 (8.6)	45 (7.0)	0.046
CVD	125 (1.7)	93 (1.5)	10 (2.1)	22 (3.3)	0.001		97 (1.5)	13 (2.7)	15 (2.3)	0.060

Categorical variables are expressed as numbers (%), and continuous variables are expressed as mean ± standard deviation

BMI, body mass index; COPD, chronic obstructive pulmonary disease; CVD, cardiovascular disease; DM, diabetes mellitus; FEV_1_, forced expiratory volume in 1 s; FVC, functional vital capacity; HTN, hypertension; PRISm, preserved ratio of impaired spirometry

### All-cause mortality risk of PRISm

During the 16.5-year mean follow-up period, 113 (16.9%) deaths occurred in the COPD group, 40 (8.5%) in the PRISm group, and 347 (5.4%) in the normal group according to the FR criteria. Kaplan-Meier analysis showed that the survival curve of the PRISm group was intermediate between those of the normal and COPD groups (Supplementary Figure S1). After classifying patients with COPD according to the Global Initiative on Obstructive Lung Disease (GOLD) stage, the survival curve of the PRISm group was between those of the normal and COPD GOLD stage 1 groups (Supplementary Figure S2). In the multivariable Cox proportional hazards regression, PRISm was not a significant predictor of all-cause mortality, whereas COPD was a significant predictor of all-cause mortality after adjusting for age, sex, BMI, smoking status, and comorbidities, including hypertension (HTN), diabetes mellitus (DM), and cardiovascular disease (CVD) (Table 2). Other variables, including male sex, old age, DM, and current smoking status, were also associated with increased mortality in the multivariable analysis. According to the LLN criteria, 90 (14.0%) deaths occurred in the COPD group, 45 (9.2%) in the PRISm group, and 365 (5.7%) in the control group during the follow-up period. The results of the analyses of all-cause mortality risk of PRISm according to the LLN criterion did not differ from those of PRISm according to the FR criterion (Supplementary Table S1).

**Table 2:** Multivariable Cox proportional analysis for predicting all-cause mortality

	aHR	95% CI
Group^a^		
Normal	Ref.	
PRISm	1.193	0.859–1.658
COPD	1.347	1.070–1.695
Sex		
Female	Ref.	
Male	1.494	1.134–1.969
Age group	1.116	1.103–1.130
BMI	0.975	0.946–1.005
Smoking status		
Never	Ref.	
Former	1.288	0.943–1.759
Current	1.607	1.212–2.130
HTN	0.910	0.728–1.138
DM	2.155	1.689–2.754
CVD	1.414	0.875–2.284

^a^Airflow limitation is defined using a fixed-ratio criterion

aHR, adjusted hazard ratio; BMI, body mass index; CI, confidence interval; COPD, chronic obstructive pulmonary disease; DM, diabetes mellitus; HTN, hypertension; PRISm, preserved ratio of impaired spirometry

### Cardiovascular mortality risk of PRISm

Of the 500 deaths, 106 cardiovascular deaths were observed during the 16.5-year mean follow-up period. Kaplan-Meier analysis showed that the cardiovascular mortality risk was higher in the COPD and PRISm groups than in the normal group (Supplementary Figure S3). In the multivariable Cox proportional hazards regression, COPD and PRISm were significant predictors of cardiovascular mortality after adjusting for age, sex, BMI, smoking status, and comorbidities, including HTN, DM, and CVD. However, according to the LLN criteria, PRISm was not a significant predictor of cardiovascular mortality, whereas COPD remained a significant predictor of cardiovascular mortality after adjusting for the variables (Table 3).

**Table 3:** Multivariable Cox proportional analysis for predicting cardiovascular mortality

	Fixed ratio		Lower limit of normal		
	aHR	95% CI		aHR	95% CI
Group					
Normal	Ref.			Ref.	
PRISm	1.983	1.088–3.614		1.653	0.925–2.953
COPD	1.696	1.041–2.761		1.833	1.094–3.070
Sex					
Female	Ref.			Ref.	
Male	0.933	0.513–1.697		1.000	0.551–1.817
Age group	1.156	1.121–1.192		1.159	1.124–1.194
BMI	0.973	0.911–1.040		0.973	0.910–1.040
Smoking status					
Never	Ref.			Ref.	
Former	1.546	0.779–3.068		1.500	0.753–2.985
Current	1.698	0.906–3.182		1.633	0.867–3.074
HTN	0.860	0.543–1.361		0.853	0.539–1.349
DM	0.311	0.196–0.496		0.313	0.197–0.498
CVD	2.283	1.031–5.056		2.314	1.050–5.100

aHR, adjusted hazard ratio; BMI, body mass index; CI, confidence interval; COPD, chronic obstructive pulmonary disease; CVD, cardiovascular disease; DM, diabetes mellitus; HTN, hypertension; PRISm, preserved ratio of impaired spirometry

## Discussion

In the present study, we evaluated the prognosis of PRISm patients compared with that of COPD patients and normal individuals in the general Asian population. In the present study, the prevalence of PRISm was 6.2% according to the FR criteria and 6.4% according to the LLN criteria. We found that those with PRISm did not have a significantly increased risk of all-cause mortality compared with individuals with normal lung function. Those with PRISm showed a significantly increased cardiovascular mortality risk compared with individuals with normal lung function, which, however, lost significance when participants were classified according to the LLN criterion.

Our results are different from those of the Rotterdam and COPDGene studies, which found increased all-cause mortality in PRISm patients compared with COPD GOLD 2–4 patients [10, 11]. The different results in the present study stem from several factors. First, the mean age of the PRISm group in previous studies was 10 years older than that in the present study. Most studies have investigated mortality risk in older PRISm [4, 7, 10, 25]. Although the COPD gene study and the TESAOD cohort enrolled younger participants than other studies, the mean ages were older than those in the present study [2, 25]. In the present study, more than 40% of patients in the PRISm group were between 40 and 49 years old; to the best of our knowledge, this is the youngest PRISm group. Second, the PRISm group in the present study included a lower proportion of smokers than in other studies. In the COPD gene study, all participants were ever-smokers, and the Rotterdam study showed that only approximately 30% of the PRISm group were never smokers. In the present study, 55.2% of the patients in the PRISm group were never smokers, which is in line with the proportion of never smokers in the PRISm group previously reported in Korean and Japanese general population-based data [6, 26]. This is likely due to the lower smoking rate among women in Asian populations than in Western population [27, 28]. Third, in the present study, although the PRISm group had more participants with a higher BMI than the other groups, compared with previous studies conducted in Western countries, the absolute value of BMI in the PRISm group (mean, 24.9 kg/m^2^) was low, and the differences with other groups were not large [2, 10, 22]. This is in line with previous studies conducted in Asian countries, which were predictable considering the characteristics of Asian populations [6, 13, 29].

Although few studies have investigated the cause-specific mortality associated with PRISm, several studies have consistently confirmed the increased cardiovascular mortality risk associated with PRISm [2, 6, 10, 29]. The increased all-cause mortality risk of PRISm seems to be attributable to increased cardiovascular mortality risk [2, 6, 29]. Interestingly, cardiovascular mortality did not increase in this study. When classified according to the FR criterion, the PRISm group showed a significantly higher risk than the normal group; however, this difference disappeared when the LLN criterion was used. FR does not reflect the normal cutoff for the FEV1/FVC ratio, which declines with age, leading to an excessive diagnosis of COPD among older people and an insufficient diagnosis among young people [30–32]. It has also been reported that LLN predicts the prognosis of COPD patients better than FR especially in middle-aged adults [31–33]. Considering the small number of older participants in the present study, the results based on LLN may better reflect the actual cardiovascular risk of PRISm in our population. The exact mechanisms underlying cardiovascular risk and PRISm have not been fully elucidated; however, several hypotheses have been proposed. Cardiovascular comorbidities and obesity are suggested as contributing factors to the restrictive lung function abnormality of PRISm, leading to increased cardiovascular mortality [3, 4, 8]. Characteristics such as young age, low proportion of smokers, and low BMI of our PRISm population could explain the cardiovascular mortality results of our study because these are also well-known risk factors for cardiovascular mortality. Moreover, in our study, the baseline prevalence of HTN, DM, and CVD was not significantly higher in the PRISm group than in the other groups. Therefore, the PRISm population in our study was suggested to differ from other PRISm populations in previous studies. PRISm comprises a collection of diverse and heterogeneous characteristics [5, 15]. In the COPDGene study, investigators hypothesized that there would be a higher- or lower-risk subgroup in the PRISm group and confirmed the various transitions of the subgroups by examining longitudinal follow-ups [10, 11]. Given the high prevalence of pulmonary tuberculosis in South Korea and the large proportion of light smokers in our PRISm population, the etiology of many PRISm cases included in this study may be due to lung impairment caused by tuberculosis infection or asthma [26, 34, 35]. The trajectory of PRISm in various populations, including light smokers and Asians or young populations, is needed. Further studies are needed to distinguish between the higher- and lower-risk groups in the PRISm population.

The main strength of our study is the relatively large sample size of the Asian population, including a considerable proportion of middle-aged individuals, which is different from other studies, as well as a population-based setting. Other strengths include a long-term follow-up period, availability of cause-specific mortality analyses, use of the exact definition of PRISm, and analysis based on the LLN criterion. The use of pre-bronchodilator spirometry to assess AFL is a limitation of the present study. Assessing AFL using post-bronchodilator spirometry could more accurately determine the prognosis of patients with PRISm and COPD by selecting reversibility.

## Conclusion

We confirmed that all-cause mortality risk and cardiovascular mortality risk are not always increased in individuals with PRISm if the population has certain characteristics, such as middle-aged light smokers without additional cardiovascular risk. Further studies are needed to distinguish lower-risk subgroups within PRISm.

## Electronic supplementary material

Below is the link to the electronic supplementary material.

Supplementary Material 1: **Supplementary figure S1**. All-cause mortality risk of PRISm group compared to COPD and normal groups. **Supplementary figure S2** All-cause mortality risk of PRISm group compared to COPD GOLD stages and normal groups. **Supplementary table S1** Multivariable cox proportional analysis for predicting all-cause mortality. **Supplementary figure S3** Cardiovascular mortality risk of PRISm group compared to COPD and normal groups

## Data Availability

The datasets used and/or analyzed in the current study are available from the corresponding author upon reasonable request.

## Abbreviations

aHR: adjusted hazard ratio.
AFL: airflow limitation.
BMI: body mass index.
COPD: chronic obstructive pulmonary disease.
CI: confidence interval.
CVD: cardiovascular disease.
DM: diabetes mellitus.
FR: fixed ratio.
FEV_1_: forced expiratory volume in 1 s.
FVC: forced vital capacity.
GOLD: Global Initiative on Obstructive Lung Disease.
HTN: hypertension.
KoGES: Korean Genome and Epidemiology Study.
LLN: lower limit of normal.
PRISm: preserved ratio impaired spirometry.
