# Supplementary material for: Longitudinal mortality of preserved ratio impaired spirometry in a middle-aged Asian cohort
Source: BMC Pulm Med. 2023 May 3;23:155. doi: 10.1186/s12890-023-02451-2 (PMC10157957; doi:10.1186/s12890-023-02451-2)
Supplement: Supplementary file 1 — Supplementary Material 1: Supplementary figure S1. All-cause mortality risk of PRISm group compared to COPD and normal groups. Supplementary figure S2 All-cause mortality risk of PRISm group compared to COPD GOLD stages and normal groups. Supplementary table S1 Multivariable cox proportional analysis for predicting all-cause mortality. Supplementary figure S3 Cardiovascular mortality risk of PRISm group compared to COPD and normal groups [file 12890_2023_2451_MOESM1_ESM.docx]

**Longitudinal Mortality of Preserved Ratio Impaired Spirometry in middle-aged Asian cohort**

Sooim Sin^1^, Eun Joo Lee^2^, Sungho Won^3,4^, Woo Jin Kim^2,5*^

^1^Division of Pulmonary and Critical Care Medicine, Department of Internal Medicine, National Medical Center, Seoul, Republic of Korea

^2^Department of Internal Medicine and Environmental Health Center, Kangwon National University Hospital, Chuncheon, Republic of Korea

^3^Interdisciplinary Program in Bioinformatics, Seoul National University, Seoul, Republic of Korea

^4^Department of Public Health Science, Graduate School of Public Health, Seoul National University, Seoul, Republic of Korea

^5^Department of Internal Medicine, School of Medicine, Kangwon National University, Chuncheon, Republic of Korea

***Correspondence author:** Woo Jin Kim, MD, PhD

Department of Internal Medicine, School of Medicine, Kangwon National University, Kangwon National University Hospital, Chuncheon 24341, Republic of Korea, Tel: +82-33-258-9364; Fax: +82-33-258-2404; E-mail: [wjkim47@gmail.com](mailto:wjkim47@gmail.com)

**Supplementary Figure S1.** All-cause mortality risk of PRISm group compared to COPD and normal groups





**Supplementary Figure S2.** All-cause mortality risk of PRISm group compared to COPD GOLD stages and normal groups


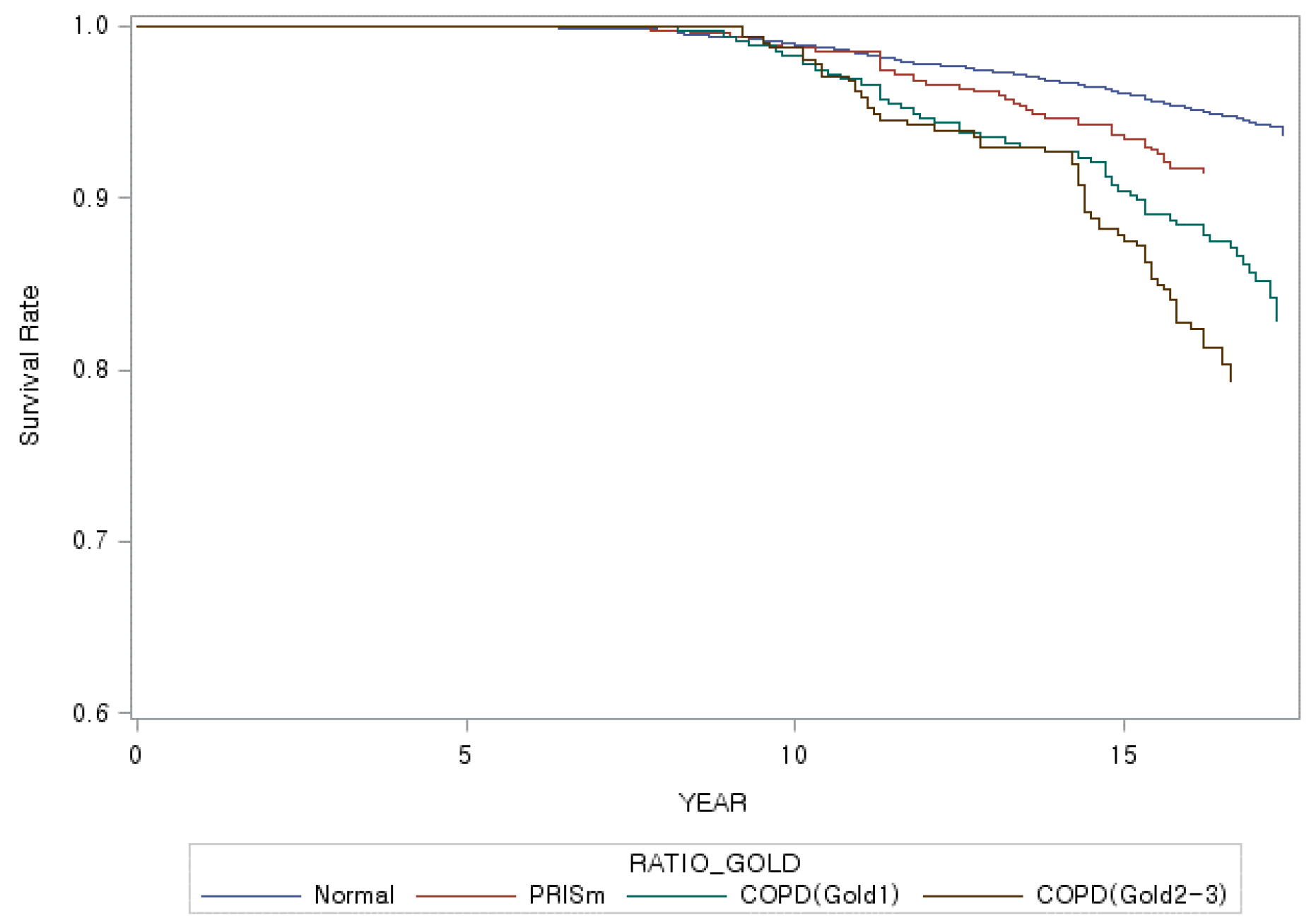


**Supplementary Table S1.** Multivariable cox proportional analysis for predicting all-cause mortality

|  | aHR | 95% CI |
| --- | --- | --- |
| Group^*^ |  |  |
| Normal | ref. | . |
| PRISm | 1.072 | 0.784-1.466 |
| COPD | 1.599 | 1.259-2.031 |
| Sex |  |  |
| Female | ref. | . |
| Male | 1.543 | 1.172-2.031 |
| Age group | 1.118 | 1.105-1.131 |
| BMI, kg/m^2^ | 0.976 | 0.946-1.006 |
| Smoking state |  |  |
| Never | ref. | . |
| Former | 1.265 | 0.926-1.728 |
| Current | 1.563 | 1.179-2.074 |
| Hypertension | 0.902 | 0.721-1.128 |
| Diabetes | 2.154 | 1.690-2.757 |
| Cardiovascular disease | 1.424 | 0.883-2.298 |

^*^Airflow limitation was defined by the lower limit of normal criterion.

CI, confidence interval; BMI, body mass index; COPD, chronic obstructive pulmonary disease; PRISm, preserved ratio impaired spirometry.

**Supplementary Figure S3.** Cardiovascular mortality risk of PRISm group compared to COPD and normal groups
